# Supplementary material for: In vivo transcriptomic profiling using cell encapsulation identifies effector pathways of systemic aging
Source: eLife. 2022 Mar 4;11:e57393. doi: 10.7554/eLife.57393 (PMC8926399; doi:10.7554/eLife.57393)
Supplement: Supplementary file 1. — (a) Multiplexed enzyme-linked immunosorbent assay (ELISA) quantification of inflammatory factors in serum of mice that underwent surgical implantation of polyethersulfone (PES) hollow fiber capsules compared to untreated controls 10 days after the procedure. n ≥ 7 mice. Each value represents averages in pg/ml from n = 4 technical replicates for each factor. TNF-Rl and MIP-1g were excluded from the analysis since signals were outside of the detection range. (b) Gene sets increased with age in encapsulated human skeletal muscle progenitors (hskMPs) compared to the young control after 10 days in vivo. Data are derived from capsules of n ≥ 5 mice in each age group. GST p-value (Pval) = Wilcoxon gene set test p-value. GST FDR = Adjusted p-value using the Benjamini-Hochberg procedure. GST FDR = Wilcoxon gene set test false discovery rate. (c) Gene sets increased with age in encapsulated mouse skeletal muscle progenitors (mskMPs) compared to the young control after 10 days in vivo. Data are derived from capsules of n ≥ 5 mice in each age group. GST Pval = Wilcoxon gene set test p-value. GST FDR = Adjusted p-value using the Benjamini-Hochberg procedure. (d) Significantly enriched Hallmark Myc V1 target genes when comparing encapsulated hskMPs in aged mice to the young control after 10 days in vivo. Data are derived from capsules of n ≥ 5 mice in each age group. Pval < 1%. (e) Significantly enriched Hallmark E2F target genes when comparing encapsulated hskMPs in aged mice to the young control after 10 days in vivo. Data are derived from capsules of n ≥ 5 mice in each age group. Pval < 1%. (f) Multiplexed ELISA array quantification of levels of inflammatory factors in plasma of mice young and aged mice. n ≥ 5 mice. Each value represents averages in pg/ml from n = 4 technical replicates for each factor. TNF-Rl and MIP-1g were excluded from the analysis since signals were outside of the detection range. (g) Gene sets decreased with age in encapsulated hskMPs compared to the you [file elife-57393-supp1.doc]

*In Vivo* Transcriptomic Profiling using Cell Encapsulation Identifies Effector Pathways of Systemic Aging

Omid Mashinchian, Xiaotong Hong, Joris Michaud, Eugenia Migliavacca, Gregory Lefebvre, Christophe Boss, Filippo De Franceschi, Emmeran Le Moal, Jasmin Collerette-Tremblay, Joan Isern, Sylviane Metairon, Frederic Raymond, Patrick Descombes, Nicolas Bouche, Pura Muñoz-Cánoves, Jerome N. Feige, C. Florian Bentzinger

Supplementary Information

Supplementary files 1a-n

|  | **-Capsule** | | | | | | | **+Capsule** | | | | | | | |
| --- | --- | --- | --- | --- | --- | --- | --- | --- | --- | --- | --- | --- | --- | --- | --- |
| **Mouse** | **1** | **2** | **3** | **4** | **5** | **6** | **7** | **1** | **2** | **3** | **4** | **5** | **6** | **7** | **8** |
| **BLC** | 18.5 | 28.5 | 20.5 | 14.4 | 23.0 | 18.6 | 18.2 | 33.6 | 16.7 | 16.3 | 21.3 | 21.2 | 15.1 | 24.0 | 15.1 |
| **CD30L** | 11.2 | 9.4 | 13.9 | 1.1 | 5.4 | 13.3 | 8.2 | 35.2 | 3.1 | 13.2 | 6.7 | 15.2 | 6.9 | 21.8 | 18.8 |
| **Eotaxin** | 312.8 | 344.3 | 183.5 | 240.3 | 122.8 | 159.1 | 302.6 | 213.1 | 139.2 | 181.6 | 92.7 | 253.5 | 250.7 | 245.6 | 192.2 |
| **Eotaxin-2** | 165.7 | 212.9 | 96.7 | 63.6 | 80.9 | 107.4 | 118.4 | 125.8 | 66.0 | 71.5 | 72.6 | 57.8 | 52.9 | 153.6 | 78.4 |
| **Fas L** | 115.4 | 17.8 | 30.8 | 13.0 | 36.4 | 119.8 | 92.0 | 100.6 | 11.1 | 77.1 | 25.6 | 0.0 | 1.6 | 6.0 | 9.1 |
| **G-CSF** | 219.4 | 168.9 | 113.9 | 126.1 | 160.7 | 145.1 | 186.4 | 211.0 | 156.4 | 229.1 | 255.7 | 62.7 | 118.5 | 130.1 | 145.6 |
| **GM-CSF** | 26.2 | 20.3 | 21.3 | 19.2 | 9.9 | 21.7 | 20.6 | 16.1 | 12.6 | 12.0 | 16.2 | 27.0 | 19.2 | 27.2 | 25.3 |
| **ICAM-1** | 1873.4 | 2621.3 | 2061.8 | 2268.4 | 1723.5 | 1824.2 | 1947.8 | 1765.7 | 1440.2 | 1896.0 | 1588.8 | 1871.7 | 1597.9 | 2409.4 | 1874.0 |
| **IFNg** | 66.7 | 65.9 | 63.8 | 85.6 | 39.0 | 69.7 | 51.2 | 67.5 | 25.4 | 40.0 | 43.0 | 68.1 | 78.2 | 85.5 | 65.4 |
| **IL-1a** | 4.6 | 17.0 | 5.3 | 8.4 | 2.7 | 4.4 | 4.6 | 3.0 | 3.8 | 1.6 | 2.9 | 8.0 | 3.3 | 4.8 | 4.8 |
| **IL-1b** | 171.3 | 164.0 | 143.3 | 183.4 | 77.5 | 149.3 | 123.9 | 156.0 | 72.4 | 83.5 | 80.2 | 175.8 | 167.0 | 185.9 | 162.6 |
| **IL-2** | 116.4 | 91.6 | 77.9 | 116.6 | 57.5 | 106.1 | 122.5 | 88.0 | 74.8 | 84.9 | 69.0 | 123.7 | 114.0 | 121.2 | 97.2 |
| **IL-3** | 3.8 | 1.1 | 1.5 | 0.8 | 2.6 | 2.7 | 3.3 | 1.8 | 0.3 | 1.4 | 1.1 | 2.4 | 1.5 | 1.4 | 1.7 |
| **IL-4** | 2.2 | 0.0 | 2.8 | 2.3 | 1.9 | 1.6 | 1.1 | 3.6 | 0.8 | 1.7 | 1.9 | 1.5 | 1.1 | 0.8 | 1.3 |
| **IL-5** | 131.3 | 71.0 | 79.2 | 69.2 | 66.4 | 111.7 | 156.0 | 91.7 | 43.6 | 75.2 | 48.7 | 92.8 | 69.7 | 59.0 | 92.5 |
| **IL-6** | 23.6 | 19.5 | 11.3 | 16.4 | 14.4 | 25.3 | 25.8 | 11.9 | 3.0 | 17.9 | 9.7 | 17.0 | 14.7 | 14.6 | 15.9 |
| **IL-7** | 87.3 | 46.8 | 0.0 | 9.5 | 21.8 | 56.3 | 10.0 | 116.5 | 65.8 | 3.6 | 27.9 | 38.0 | 0.0 | 65.7 | 194.5 |
| **IL-10** | 324.7 | 307.3 | 261.2 | 272.0 | 119.3 | 262.4 | 309.6 | 287.5 | 139.5 | 147.3 | 142.0 | 340.5 | 292.4 | 316.5 | 323.1 |
| **IL-12p70** | 93.2 | 56.1 | 72.5 | 62.3 | 38.2 | 85.3 | 96.0 | 100.3 | 37.2 | 65.8 | 36.6 | 96.6 | 84.2 | 107.1 | 88.8 |
| **IL-13** | 171.3 | 6.2 | 18.5 | 4.8 | 4.5 | 0.0 | 0.0 | 31.4 | 0.0 | 0.9 | 6.4 | 0.0 | 0.0 | 3.1 | 0.0 |
| **IL-15** | 479.5 | 136.3 | 265.4 | 107.0 | 194.8 | 360.8 | 161.7 | 482.5 | 138.8 | 202.3 | 153.6 | 105.6 | 145.8 | 81.5 | 168.7 |
| **IL-17** | 2.1 | 0.1 | 4.0 | 1.2 | 3.6 | 3.5 | 4.1 | 3.6 | 1.7 | 2.7 | 0.9 | 2.0 | 1.2 | 3.6 | 1.8 |
| **IL-21** | 5.7 | 5.6 | 0.0 | 0.0 | 5.8 | 8.5 | 1.8 | 15.1 | 14.4 | 4.0 | 0.0 | 0.0 | 0.0 | 15.5 | 20.1 |
| **KC** | 4.6 | 4.8 | 3.6 | 4.4 | 2.0 | 3.3 | 4.4 | 3.7 | 2.9 | 2.9 | 2.7 | 3.9 | 3.5 | 3.9 | 3.2 |
| **Leptin** | 661.5 | 586.4 | 665.4 | 296.8 | 428.9 | 414.8 | 432.2 | 140.3 | 91.7 | 188.0 | 105.9 | 161.8 | 78.7 | 83.4 | 161.9 |
| **LIX** | 508.7 | 553.1 | 377.6 | 460.3 | 215.1 | 379.6 | 153.0 | 356.0 | 322.0 | 292.9 | 230.1 | 531.1 | 381.0 | 482.4 | 392.8 |
| **MCP-1** | 59.6 | 30.9 | 24.2 | 29.9 | 15.9 | 30.2 | 42.2 | 45.0 | 20.3 | 21.4 | 16.8 | 37.7 | 32.9 | 45.0 | 40.9 |
| **MCP-5** | 47.9 | 32.3 | 36.4 | 21.6 | 29.4 | 30.1 | 18.9 | 53.2 | 25.8 | 19.8 | 11.6 | 40.9 | 20.9 | 11.1 | 31.4 |
| **MCSF** | 9.6 | 0.7 | 1.9 | 3.4 | 4.1 | 7.2 | 2.6 | 4.3 | 0.8 | 6.1 | 3.7 | 1.5 | 3.1 | 2.0 | 1.7 |
| **MIG** | 41.0 | 49.3 | 45.0 | 40.9 | 42.3 | 43.7 | 46.2 | 67.0 | 35.5 | 25.7 | 28.1 | 36.9 | 31.8 | 32.8 | 40.9 |
| **MIP-1a** | 24.1 | 16.1 | 13.9 | 12.2 | 11.6 | 36.7 | 16.8 | 8.4 | 10.3 | 19.6 | 8.9 | 12.8 | 12.6 | 10.8 | 12.5 |
| **PF4** | 3066.6 | 3415.4 | 3692.6 | 3006.8 | 5273.3 | 3779.9 | 2905.3 | 3734.5 | 4274.5 | 4317.3 | 4222.6 | 3164.9 | 2978.8 | 3612.0 | 3644.5 |
| **RANTES** | 3.2 | 2.6 | 1.7 | 1.8 | 1.5 | 4.3 | 3.3 | 1.7 | 1.3 | 2.2 | 1.4 | 2.1 | 1.8 | 1.5 | 1.8 |
| **TARC** | 40.9 | 48.3 | 26.0 | 31.1 | 18.4 | 26.9 | 23.0 | 35.2 | 14.0 | 19.5 | 12.1 | 30.0 | 20.1 | 36.6 | 20.7 |
| **TCA-3** | 18.0 | 19.5 | 21.4 | 14.9 | 21.0 | 26.6 | 11.1 | 34.7 | 23.5 | 23.3 | 10.4 | 45.4 | 14.2 | 38.8 | 30.2 |
| **TIMP-1** | 597.3 | 575.2 | 638.2 | 442.1 | 431.9 | 456.9 | 512.9 | 721.0 | 479.6 | 587.1 | 472.9 | 563.8 | 544.4 | 555.8 | 445.5 |
| **TNFa** | 29.8 | 37.4 | 41.9 | 48.4 | 17.0 | 43.1 | 34.8 | 28.0 | 19.2 | 18.2 | 22.7 | 44.8 | 34.4 | 42.6 | 44.5 |
| **TNF RII** | 412.8 | 370.1 | 350.4 | 196.1 | 348.9 | 267.6 | 116.9 | 416.2 | 209.0 | 373.4 | 259.0 | 396.6 | 367.6 | 306.4 | 113.3 |

**Supplementary file 1a |** Multiplexed ELISA quantification of inflammatory factors in serum of mice that underwent surgical implantation of PES hollow fiber capsules compared to untreated controls ten days after the procedure. n≥7 mice. Each value represents averages in pg/ml from n=4 technical replicates for each factor. TNF-Rl and MIP-1g were excluded from the analysis since signals were outside of the detection range.

| **Gene Set Name - Human** | **GST Pval** | **GST FDR** |
| --- | --- | --- |
| HALLMARK_MYC_TARGETS_V1 | 1.00E-04 | 0.005 |
| HALLMARK_OXIDATIVE_PHOSPHORYLATION | 2.00E-04 | 0.005 |
| HALLMARK_E2F_TARGETS | 0.0166 | 0.238 |
| HALLMARK_FATTY_ACID_METABOLISM | 0.0215 | 0.238 |
| HALLMARK_PANCREAS_BETA_CELLS | 0.0238 | 0.238 |
| HALLMARK_ANGIOGENESIS | 0.0648 | 0.506428571 |
| HALLMARK_COAGULATION | 0.0709 | 0.506428571 |
| HALLMARK_KRAS_SIGNALING_UP | 0.1885 | 1 |
| HALLMARK_XENOBIOTIC_METABOLISM | 0.1988 | 1 |
| HALLMARK_ADIPOGENESIS | 0.2427 | 1 |
| HALLMARK_G2M_CHECKPOINT | 0.2755 | 1 |
| HALLMARK_REACTIVE_OXIGEN_SPECIES_PATHWAY | 0.284 | 1 |
| HALLMARK_SPERMATOGENESIS | 0.3227 | 1 |
| HALLMARK_ANDROGEN_RESPONSE | 0.3554 | 1 |
| HALLMARK_WNT_BETA_CATENIN_SIGNALING | 0.3626 | 1 |
| HALLMARK_PROTEIN_SECRETION | 0.4046 | 1 |
| HALLMARK_CHOLESTEROL_HOMEOSTASIS | 0.5342 | 1 |
| HALLMARK_MYC_TARGETS_V2 | 0.633 | 1 |
| HALLMARK_BILE_ACID_METABOLISM | 0.6826 | 1 |
| HALLMARK_HEME_METABOLISM | 0.7257 | 1 |
| HALLMARK_TGF_BETA_SIGNALING | 0.7263 | 1 |
| HALLMARK_PEROXISOME | 0.7279 | 1 |
| HALLMARK_DNA_REPAIR | 0.7765 | 1 |
| HALLMARK_ALLOGRAFT_REJECTION | 0.788 | 1 |
| HALLMARK_MTORC1_SIGNALING | 0.9169 | 1 |
| HALLMARK_UV_RESPONSE_UP | 0.9296 | 1 |
| HALLMARK_APICAL_SURFACE | 0.9408 | 1 |
| HALLMARK_HEDGEHOG_SIGNALING | 0.9426 | 1 |
| HALLMARK_ESTROGEN_RESPONSE_LATE | 0.9434 | 1 |
| HALLMARK_IL2_STAT5_SIGNALING | 0.961 | 1 |
| HALLMARK_GLYCOLYSIS | 0.963 | 1 |
| HALLMARK_COMPLEMENT | 0.964 | 1 |
| HALLMARK_UV_RESPONSE_DN | 0.9709 | 1 |
| HALLMARK_APOPTOSIS | 0.9756 | 1 |
| HALLMARK_EPITHELIAL_MESENCHYMAL_TRANSITION | 0.979 | 1 |
| HALLMARK_INTERFERON_ALPHA_RESPONSE | 0.9823 | 1 |
| HALLMARK_UNFOLDED_PROTEIN_RESPONSE | 0.9841 | 1 |
| HALLMARK_APICAL_JUNCTION | 0.9851 | 1 |
| HALLMARK_NOTCH_SIGNALING | 0.9871 | 1 |
| HALLMARK_P53_PATHWAY | 0.9898 | 1 |
| HALLMARK_MITOTIC_SPINDLE | 0.9913 | 1 |
| HALLMARK_IL6_JAK_STAT3_SIGNALING | 0.992 | 1 |
| HALLMARK_PI3K_AKT_MTOR_SIGNALING | 0.9961 | 1 |
| HALLMARK_INTERFERON_GAMMA_RESPONSE | 0.9976 | 1 |
| HALLMARK_KRAS_SIGNALING_DN | 0.9978 | 1 |
| HALLMARK_HYPOXIA | 0.9986 | 1 |
| HALLMARK_INFLAMMATORY_RESPONSE | 0.9988 | 1 |
| HALLMARK_ESTROGEN_RESPONSE_EARLY | 0.9996 | 1 |
| HALLMARK_TNFA_SIGNALING_VIA_NFKB | 0.9998 | 1 |
| HALLMARK_MYOGENESIS | 1 | 1 |

**Supplementary file 1b |** Gene sets increased with age in encapsulated hskMPs compared to the young control after ten days *in vivo*. Data are derived from capsules of n≥5 mice in each age group. GST Pval = Wilcoxon gene set test p- value. GST FDR = Adjusted p-value using the Benjamini-Hochberg procedure. GST FDR = Wilcoxon gene set test false discovery rate.

| **Gene Set Name - Mouse** | **GST Pval** | **GST FDR** |
| --- | --- | --- |
| HALLMARK_MITOTIC_SPINDLE | 1.00E-04 | 0.00125 |
| HALLMARK_G2M_CHECKPOINT | 1.00E-04 | 0.00125 |
| HALLMARK_E2F_TARGETS | 1.00E-04 | 0.00125 |
| HALLMARK_MYC_TARGETS_V1 | 1.00E-04 | 0.00125 |
| HALLMARK_UV_RESPONSE_DN | 0.0271 | 0.245833333 |
| HALLMARK_MYC_TARGETS_V2 | 0.0295 | 0.245833333 |
| HALLMARK_HEDGEHOG_SIGNALING | 0.0512 | 0.365714286 |
| HALLMARK_CHOLESTEROL_HOMEOSTASIS | 0.1214 | 0.677777778 |
| HALLMARK_KRAS_SIGNALING_DN | 0.122 | 0.677777778 |
| HALLMARK_NOTCH_SIGNALING | 0.1655 | 0.8275 |
| HALLMARK_TGF_BETA_SIGNALING | 0.2272 | 1 |
| HALLMARK_APICAL_JUNCTION | 0.3936 | 1 |
| HALLMARK_PI3K_AKT_MTOR_SIGNALING | 0.4044 | 1 |
| HALLMARK_ANDROGEN_RESPONSE | 0.4386 | 1 |
| HALLMARK_FATTY_ACID_METABOLISM | 0.5445 | 1 |
| HALLMARK_OXIDATIVE_PHOSPHORYLATION | 0.5468 | 1 |
| HALLMARK_MTORC1_SIGNALING | 0.6076 | 1 |
| HALLMARK_ESTROGEN_RESPONSE_LATE | 0.653 | 1 |
| HALLMARK_SPERMATOGENESIS | 0.6719 | 1 |
| HALLMARK_TNFA_SIGNALING_VIA_NFKB | 0.712 | 1 |
| HALLMARK_APICAL_SURFACE | 0.7926 | 1 |
| HALLMARK_INTERFERON_ALPHA_RESPONSE | 0.7937 | 1 |
| HALLMARK_UNFOLDED_PROTEIN_RESPONSE | 0.8104 | 1 |
| HALLMARK_APOPTOSIS | 0.8208 | 1 |
| HALLMARK_PEROXISOME | 0.823 | 1 |
| HALLMARK_HYPOXIA | 0.8294 | 1 |
| HALLMARK_WNT_BETA_CATENIN_SIGNALING | 0.8509 | 1 |
| HALLMARK_DNA_REPAIR | 0.8616 | 1 |
| HALLMARK_UV_RESPONSE_UP | 0.8777 | 1 |
| HALLMARK_ESTROGEN_RESPONSE_EARLY | 0.8856 | 1 |
| HALLMARK_GLYCOLYSIS | 0.8878 | 1 |
| HALLMARK_EPITHELIAL_MESENCHYMAL_TRANSITION | 0.8937 | 1 |
| HALLMARK_PROTEIN_SECRETION | 0.9015 | 1 |
| HALLMARK_MYOGENESIS | 0.9268 | 1 |
| HALLMARK_BILE_ACID_METABOLISM | 0.9381 | 1 |
| HALLMARK_PANCREAS_BETA_CELLS | 0.9405 | 1 |
| HALLMARK_ALLOGRAFT_REJECTION | 0.9481 | 1 |
| HALLMARK_HEME_METABOLISM | 0.9549 | 1 |
| HALLMARK_INTERFERON_GAMMA_RESPONSE | 0.9629 | 1 |
| HALLMARK_INFLAMMATORY_RESPONSE | 0.9704 | 1 |
| HALLMARK_ANGIOGENESIS | 0.9902 | 1 |
| HALLMARK_IL6_JAK_STAT3_SIGNALING | 0.9955 | 1 |
| HALLMARK_REACTIVE_OXIGEN_SPECIES_PATHWAY | 0.9964 | 1 |
| HALLMARK_ADIPOGENESIS | 0.9986 | 1 |
| HALLMARK_COMPLEMENT | 0.9992 | 1 |
| HALLMARK_P53_PATHWAY | 0.9999 | 1 |
| HALLMARK_XENOBIOTIC_METABOLISM | 1 | 1 |
| HALLMARK_COAGULATION | 1 | 1 |
| HALLMARK_IL2_STAT5_SIGNALING | 1 | 1 |
| HALLMARK_KRAS_SIGNALING_UP | 1 | 1 |

**Supplementary file 1c |** Gene sets increased with age in encapsulated mskMPs compared to the young control after ten days *in vivo*. Data are derived from capsules of n≥5 mice in each age group. GST Pval = Wilcoxon gene set test p-value. GST FDR = Adjusted p-value using the Benjamini-Hochberg procedure.

| **Gene list Name: Human Myc** | **Entrez_Gene_ID** | **Pval** |  | **Gene list Name: Human Myc** | **Entrez_Gene_ID** | **Pval** |
| --- | --- | --- | --- | --- | --- | --- |
| SNRPA1 | 6627 | 0.003392238 |  | PSMA6 | 5687 | 0.303152803 |
| EEF1B2 | 1933 | 0.005434609 |  | CDK4 | 1019 | 0.309228977 |
| RAN | 5901 | 0.006608012 |  | UBA2 | 10054 | 0.310913108 |
| HSPD1 | 3329 | 0.007032145 |  | HNRNPU | 3192 | 0.317074711 |
| RPS3 | 6188 | 0.009164852 |  | CCT3 | 7203 | 0.339467799 |
| DHX15 | 1665 | 0.013753848 |  | PHB2 | 11331 | 0.342385831 |
| PSMA4 | 5685 | 0.013889751 |  | EPRS | 2058 | 0.35703362 |
| EIF3B | 8662 | 0.020952363 |  | UBE2L3 | 7332 | 0.364117639 |
| APEX1 | 328 | 0.024259783 |  | TOMM70A | 9868 | 0.372195491 |
| PPM1G | 5496 | 0.030717253 |  | HNRPA2B1 | 3181 | 0.376637968 |
| EIF4G2 | 1982 | 0.032850738 |  | PSMB3 | 5691 | 0.387885477 |
| CLNS1A | 1207 | 0.038547487 |  | PRPF31 | 26121 | 0.39110615 |
| CNBP | 7555 | 0.041247494 |  | NOP56 | 10528 | 0.401195203 |
| H2AFZ | 3015 | 0.041308998 |  | EIF1AX | 1964 | 0.409281904 |
| GOT2 | 2806 | 0.044374131 |  | RPL14 | 9045 | 0.410832143 |
| SFRS2 | 6427 | 0.050671179 |  | RANBP1 | 5902 | 0.413335163 |
| PSMD14 | 10213 | 0.056216794 |  | GNB2L1 | 10399 | 0.414275317 |
| NCBP2 | 22916 | 0.056863238 |  | RPS10 | 6204 | 0.43780544 |
| AP3S1 | 1176 | 0.058218092 |  | SNRPD2 | 6633 | 0.446926748 |
| C1QBP | 708 | 0.059635644 |  | EIF4A1 | 1973 | 0.459292469 |
| RPL34 | 6164 | 0.065106571 |  | DDX18 | 8886 | 0.482601512 |
| PSMA1 | 5682 | 0.067628383 |  | MCM7 | 4176 | 0.489656126 |
| SRM | 6723 | 0.069279662 |  | SRPK1 | 6732 | 0.534856431 |
| LSM7 | 51690 | 0.079526429 |  | NHP2 | 55651 | 0.536197653 |
| EIF4H | 7458 | 0.089024704 |  | CBX3 | 11335 | 0.543034244 |
| HNRNPR | 10236 | 0.091514993 |  | LSM2 | 57819 | 0.588783927 |
| ACP1 | 52 | 0.093385566 |  | IMPDH2 | 3615 | 0.589423651 |
| GLO1 | 2739 | 0.096041822 |  | PRPS2 | 5634 | 0.589960777 |
| HDDC2 | 51020 | 0.10373487 |  | HDAC2 | 3066 | 0.59311393 |
| ILF2 | 3608 | 0.105062374 |  | RPS2 | 6187 | 0.594295408 |
| KPNB1 | 3837 | 0.110552543 |  | SNRPG | 6637 | 0.600540544 |
| SF3B3 | 23450 | 0.116055116 |  | SSBP1 | 6742 | 0.620666447 |
| TFDP1 | 7027 | 0.120165263 |  | CUL1 | 8454 | 0.620751457 |
| RRM1 | 6240 | 0.12146948 |  | SNRPA | 6626 | 0.620999589 |
| NME1 | 4830 | 0.124680277 |  | POLD2 | 5425 | 0.623895233 |
| RPS5 | 6193 | 0.133603961 |  | MRPS18B | 28973 | 0.628720883 |
| PRDX3 | 10935 | 0.138578212 |  | FAM120A | 23196 | 0.636059558 |
| PSMA2 | 5683 | 0.145037331 |  | GSPT1 | 2935 | 0.647862269 |
| RPLP0 | 6175 | 0.151014333 |  | PGK1 | 5230 | 0.649784957 |
| TYMS | 7298 | 0.153238502 |  | CCNA2 | 890 | 0.655805663 |
| RUVBL2 | 10856 | 0.159409647 |  | RNPS1 | 10921 | 0.657031519 |
| VDAC1 | 7416 | 0.165072978 |  | AIMP2 | 7965 | 0.668976235 |
| PRDX4 | 10549 | 0.16886725 |  | HPRT1 | 3251 | 0.719650193 |
| EXOSC7 | 23016 | 0.174912282 |  | FBL | 2091 | 0.719962808 |
| NOP16 | 51491 | 0.185175615 |  | HSP90AB1 | 3326 | 0.753094124 |
| RPL18 | 6141 | 0.185269043 |  | NCBP1 | 4686 | 0.757050736 |
| SMARCC1 | 6599 | 0.185794825 |  | USP1 | 7398 | 0.767226527 |
| COPS5 | 10987 | 0.191437874 |  | MCM5 | 4174 | 0.772826422 |
| PWP1 | 11137 | 0.192517154 |  | SLC25A3 | 5250 | 0.775933741 |
| XPO1 | 7514 | 0.197505488 |  | IFRD1 | 3475 | 0.786216915 |
| PABPC1 | 26986 | 0.208075705 |  | ODC1 | 4953 | 0.789157714 |
| XRCC6 | 2547 | 0.209376027 |  | SNRPB2 | 6629 | 0.790869565 |
| PA2G4 | 5036 | 0.221226783 |  | TCP1 | 6950 | 0.817451206 |
| LDHA | 3939 | 0.225430805 |  | CCT2 | 10576 | 0.826007656 |
| MRPL23 | 6150 | 0.244494583 |  | TUFM | 7284 | 0.832394346 |
| PHB | 5245 | 0.252734546 |  | SYNCRIP | 10492 | 0.904366833 |
| CCT7 | 10574 | 0.258988772 |  | SFRS1 | 6426 | 0.960965928 |
| RFC4 | 5984 | 0.268219724 |  | RPL6 | 6128 | 0.971733909 |
| PCBP1 | 5093 | 0.275406523 |  | HNRNPD | 3184 | 0.977363883 |
| VBP1 | 7411 | 0.290212542 |  | PSMD1 | 5707 | 0.985781608 |

**Supplementary file 1d |** Significantly enriched Hallmark Myc V1 target genes when comparing encapsulated hskMPs in aged mice to the young control after ten days *in vivo*. Data are derived from capsules of n≥5 mice in each age group. P-value (Pval) <1%.

| **Gene list Name: Human E2F** | **Entrez_Gene_ID** | **Pval** |
| --- | --- | --- |
| RAN | 5901 | 0.006608012 |
| RPA2 | 6118 | 0.014860127 |
| POLE4 | 56655 | 0.01802049 |
| CKS1B | 1163 | 0.024697221 |
| CDKN1A | 1026 | 0.032848419 |
| H2AFZ | 3015 | 0.041308998 |
| TFRC | 7037 | 0.044915398 |
| SFRS2 | 6427 | 0.050671179 |
| SLBP | 7884 | 0.087256292 |
| DCTPP1 | 79077 | 0.116609801 |
| LSMD1 | 84316 | 0.117569831 |
| NME1 | 4830 | 0.124680277 |
| PHF5A | 84844 | 0.130757893 |
| KIF2C | 11004 | 0.13211421 |
| PRDX4 | 10549 | 0.16886725 |
| XPO1 | 7514 | 0.197505488 |
| XRCC6 | 2547 | 0.209376027 |
| PA2G4 | 5036 | 0.221226783 |
| HMGA1 | 3159 | 0.222380315 |
| CSE1L | 1434 | 0.281000763 |
| MCM3 | 4172 | 0.281581876 |
| NUP205 | 23165 | 0.294058659 |
| CDK4 | 1019 | 0.309228977 |
| NASP | 4678 | 0.352631128 |
| PLK4 | 10733 | 0.355090367 |
| CBX5 | 23468 | 0.3614241 |
| RAD21 | 5885 | 0.36235764 |
| TUBG1 | 7283 | 0.369973582 |
| UNG | 7374 | 0.378476061 |
| NOP56 | 10528 | 0.401195203 |
| TUBB | 203068 | 0.40819291 |
| ILF3 | 3609 | 0.412356029 |
| RANBP1 | 5902 | 0.413335163 |
| LBR | 3930 | 0.459788183 |
| MCM7 | 4176 | 0.489656126 |
| H2AFX | 3014 | 0.492478323 |
| CDCA8 | 55143 | 0.500398232 |
| ZW10 | 9183 | 0.552227285 |
| PTTG1 | 9232 | 0.570565713 |
| BUB1B | 701 | 0.575293758 |
| RPA3 | 6119 | 0.6134248 |
| POLD2 | 5425 | 0.623895233 |
| GSPT1 | 2935 | 0.647862269 |
| MTHFD2 | 10797 | 0.765228305 |
| USP1 | 7398 | 0.767226527 |
| MCM5 | 4174 | 0.772826422 |
| CROP | 51747 | 0.794736654 |
| RFC1 | 5981 | 0.810546431 |
| LOC146909 | 146909 | 0.813877648 |
| DNMT1 | 1786 | 0.832302909 |
| PDS5B | 23047 | 0.845750177 |
| MRE11A | 4361 | 0.892357382 |
| PRPS1 | 5631 | 0.904327377 |
| SYNCRIP | 10492 | 0.904366833 |
| SNRPB | 6628 | 0.912005837 |
| SFRS1 | 6426 | 0.960965928 |
| HNRNPD | 3184 | 0.977363883 |
| C6ORF167 | 253714 | 0.989129666 |
| DDX39 | 10212 | 0.996693587 |

**Supplementary file 1e |** Significantly enriched Hallmark E2F target genes when comparing encapsulated hskMPs in aged mice to the young control after ten days *in vivo*. Data are derived from capsules of n≥5 mice in each age group. P-value (Pval) <1%.

|  | **Young** | | | | | | | | **Aged** | | | | |
| --- | --- | --- | --- | --- | --- | --- | --- | --- | --- | --- | --- | --- | --- |
| **Mouse** | **1** | **2** | **3** | **4** | **5** | **6** | **7** | **8** | **1** | **2** | **3** | **4** | **5** |
| **BLC** | 33.6 | 16.7 | 16.3 | 21.3 | 21.2 | 15.1 | 24.0 | 15.1 | 95.8 | 112.4 | 75.9 | 69.4 | 81.6 |
| **CD30L** | 35.2 | 3.1 | 13.2 | 6.7 | 15.2 | 6.9 | 21.8 | 18.8 | 37.9 | 14.3 | 19.5 | 0.4 | 3.1 |
| **Eotaxin** | 213.1 | 139.2 | 181.6 | 92.7 | 253.5 | 250.7 | 245.6 | 192.2 | 703.0 | 454.6 | 192.3 | 233.9 | 271.6 |
| **Eotaxin-2** | 125.8 | 66.0 | 71.5 | 72.6 | 57.8 | 52.9 | 153.6 | 78.4 | 329.8 | 174.2 | 75.9 | 49.2 | 22.6 |
| **Fas L** | 100.6 | 11.1 | 77.1 | 25.6 | 0.0 | 1.6 | 6.0 | 9.1 | 943.1 | 22.5 | 27.0 | 0.0 | 20.7 |
| **G-CSF** | 211.0 | 156.4 | 229.1 | 255.7 | 62.7 | 118.5 | 130.1 | 145.6 | 518.0 | 241.5 | 112.8 | 108.0 | 240.6 |
| **GM-CSF** | 16.1 | 12.6 | 12.0 | 16.2 | 27.0 | 19.2 | 27.2 | 25.3 | 35.6 | 34.6 | 16.2 | 11.6 | 24.5 |
| **ICAM-1** | 1765.7 | 1440.2 | 1896.0 | 1588.8 | 1871.7 | 1597.9 | 2409.4 | 1874.0 | 2260.4 | 5278.9 | 2393.3 | 2234.2 | 2339.1 |
| **IFNg** | 67.5 | 25.4 | 40.0 | 43.0 | 68.1 | 78.2 | 85.5 | 65.4 | 117.5 | 119.0 | 55.4 | 36.4 | 100.7 |
| **IL-1a** | 3.0 | 3.8 | 1.6 | 2.9 | 8.0 | 3.3 | 4.8 | 4.8 | 25.4 | 3.7 | 3.1 | 3.4 | 2.8 |
| **IL-1b** | 156.0 | 72.4 | 83.5 | 80.2 | 175.8 | 167.0 | 185.9 | 162.6 | 248.9 | 232.1 | 143.7 | 94.0 | 195.3 |
| **IL-2** | 88.0 | 74.8 | 84.9 | 69.0 | 123.7 | 114.0 | 121.2 | 97.2 | 170.0 | 118.8 | 74.5 | 91.7 | 99.1 |
| **IL-3** | 1.8 | 0.3 | 1.4 | 1.1 | 2.4 | 1.5 | 1.4 | 1.7 | 8.8 | 2.8 | 1.9 | 0.7 | 0.7 |
| **IL-4** | 3.6 | 0.8 | 1.7 | 1.9 | 1.5 | 1.1 | 0.8 | 1.3 | 12.1 | 2.2 | 2.9 | 0.4 | 2.4 |
| **IL-5** | 91.7 | 43.6 | 75.2 | 48.7 | 92.8 | 69.7 | 59.0 | 92.5 | 175.4 | 88.6 | 122.1 | 88.4 | 42.2 |
| **IL-6** | 11.9 | 3.0 | 17.9 | 9.7 | 17.0 | 14.7 | 14.6 | 15.9 | 43.3 | 19.2 | 10.9 | 11.4 | 28.9 |
| **IL-7** | 116.5 | 65.8 | 3.6 | 27.9 | 38.0 | 0.0 | 65.7 | 194.5 | 161.4 | 146.0 | 45.5 | 75.2 | 113.8 |
| **IL-10** | 287.5 | 139.5 | 147.3 | 142.0 | 340.5 | 292.4 | 316.5 | 323.1 | 311.4 | 358.1 | 194.9 | 207.9 | 299.1 |
| **IL-12p70** | 100.3 | 37.2 | 65.8 | 36.6 | 96.6 | 84.2 | 107.1 | 88.8 | 147.1 | 109.6 | 49.2 | 47.1 | 54.4 |
| **IL-13** | 31.4 | 0.0 | 0.9 | 6.4 | 0.0 | 0.0 | 3.1 | 0.0 | 198.2 | 59.5 | 21.9 | 0.0 | 0.0 |
| **IL-15** | 482.5 | 138.8 | 202.3 | 153.6 | 105.6 | 145.8 | 81.5 | 168.7 | 228.9 | 61.3 | 0.0 | 0.0 | 0.0 |
| **IL-17** | 3.6 | 1.7 | 2.7 | 0.9 | 2.0 | 1.2 | 3.6 | 1.8 | 14.8 | 2.2 | 3.8 | 2.0 | 9.2 |
| **IL-21** | 15.1 | 14.4 | 4.0 | 0.0 | 0.0 | 0.0 | 15.5 | 20.1 | 45.4 | 8.3 | 2.4 | 7.0 | 0.0 |
| **KC** | 3.7 | 2.9 | 2.9 | 2.7 | 3.9 | 3.5 | 3.9 | 3.2 | 4.6 | 4.8 | 3.5 | 2.8 | 3.7 |
| **Leptin** | 140.3 | 91.7 | 188.0 | 105.9 | 161.8 | 78.7 | 83.4 | 161.9 | 687.3 | 1930.5 | 220.0 | 325.6 | 84.3 |
| **LIX** | 356.0 | 322.0 | 292.9 | 230.1 | 531.1 | 381.0 | 482.4 | 392.8 | 512.4 | 663.4 | 18.0 | 257.4 | 411.3 |
| **MCP-1** | 45.0 | 20.3 | 21.4 | 16.8 | 37.7 | 32.9 | 45.0 | 40.9 | 69.6 | 17.1 | 18.5 | 17.7 | 20.2 |
| **MCP-5** | 53.2 | 25.8 | 19.8 | 11.6 | 40.9 | 20.9 | 11.1 | 31.4 | 67.1 | 15.3 | 41.5 | 12.7 | 11.6 |
| **MCSF** | 4.3 | 0.8 | 6.1 | 3.7 | 1.5 | 3.1 | 2.0 | 1.7 | 9.4 | 0.9 | 2.0 | 0.1 | 0.1 |
| **MIG** | 67.0 | 35.5 | 25.7 | 28.1 | 36.9 | 31.8 | 32.8 | 40.9 | 92.5 | 70.8 | 77.6 | 52.1 | 201.7 |
| **MIP-1a** | 8.4 | 10.3 | 19.6 | 8.9 | 12.8 | 12.6 | 10.8 | 12.5 | 32.5 | 26.3 | 17.7 | 15.1 | 2.0 |
| **PF4** | 3734.5 | 4274.5 | 4317.3 | 4222.6 | 3164.9 | 2978.8 | 3612.0 | 3644.5 | 3049.0 | 3336.7 | 4330.0 | 4125.5 | 2772.5 |
| **RANTES** | 1.7 | 1.3 | 2.2 | 1.4 | 2.1 | 1.8 | 1.5 | 1.8 | 5.7 | 5.2 | 3.1 | 2.5 | 0.1 |
| **TARC** | 35.2 | 14.0 | 19.5 | 12.1 | 30.0 | 20.1 | 36.6 | 20.7 | 44.1 | 34.6 | 21.4 | 13.3 | 16.0 |
| **TCA-3** | 34.7 | 23.5 | 23.3 | 10.4 | 45.4 | 14.2 | 38.8 | 30.2 | 40.2 | 34.9 | 63.9 | 18.7 | 25.9 |
| **TIMP-1** | 721.0 | 479.6 | 587.1 | 472.9 | 563.8 | 544.4 | 555.8 | 445.5 | 1055.8 | 851.2 | 830.4 | 505.2 | 694.6 |
| **TNFa** | 28.0 | 19.2 | 18.2 | 22.7 | 44.8 | 34.4 | 42.6 | 44.5 | 64.1 | 50.0 | 29.7 | 23.1 | 35.7 |
| **TNF RII** | 416.2 | 209.0 | 373.4 | 259.0 | 396.6 | 367.6 | 306.4 | 113.3 | 439.8 | 542.5 | 401.2 | 225.7 | 248.6 |

**Supplementary file 1f |** Multiplexed ELISA array quantification of levels of inflammatory factors in plasma of mice young and aged mice. n≥5 mice. Each value represents averages in pg/ml from n=4 technical replicates for each factor. TNF-Rl and MIP-1g were excluded from the analysis since signals were outside of the detection range.

| **Gene Set Name - Human** | **GST Pval** | **GST FDR** |
| --- | --- | --- |
| HALLMARK_TNFA_SIGNALING_VIA_NFKB | 1.00E-04 | 0.0025 |
| HALLMARK_MYOGENESIS | 1.00E-04 | 0.0025 |
| HALLMARK_ESTROGEN_RESPONSE_EARLY | 4.00E-04 | 0.006666667 |
| HALLMARK_INFLAMMATORY_RESPONSE | 0.0012 | 0.015 |
| HALLMARK_KRAS_SIGNALING_DN | 0.0022 | 0.018571429 |
| HALLMARK_HYPOXIA | 0.0026 | 0.018571429 |
| HALLMARK_INTERFERON_GAMMA_RESPONSE | 0.0026 | 0.018571429 |
| HALLMARK_PI3K_AKT_MTOR_SIGNALING | 0.004 | 0.025 |
| HALLMARK_IL6_JAK_STAT3_SIGNALING | 0.0067 | 0.037222222 |
| HALLMARK_MITOTIC_SPINDLE | 0.0082 | 0.041 |
| HALLMARK_P53_PATHWAY | 0.0112 | 0.04875 |
| HALLMARK_NOTCH_SIGNALING | 0.0117 | 0.04875 |
| HALLMARK_APICAL_JUNCTION | 0.0139 | 0.053461538 |
| HALLMARK_INTERFERON_ALPHA_RESPONSE | 0.0159 | 0.055 |
| HALLMARK_UNFOLDED_PROTEIN_RESPONSE | 0.0165 | 0.055 |
| HALLMARK_EPITHELIAL_MESENCHYMAL_TRANSITION | 0.022 | 0.06875 |
| HALLMARK_APOPTOSIS | 0.0246 | 0.072352941 |
| HALLMARK_UV_RESPONSE_DN | 0.0331 | 0.085714286 |
| HALLMARK_IL2_STAT5_SIGNALING | 0.0352 | 0.085714286 |
| HALLMARK_COMPLEMENT | 0.0353 | 0.085714286 |
| HALLMARK_GLYCOLYSIS | 0.036 | 0.085714286 |
| HALLMARK_ESTROGEN_RESPONSE_LATE | 0.0493 | 0.112045455 |
| HALLMARK_HEDGEHOG_SIGNALING | 0.0537 | 0.11673913 |
| HALLMARK_APICAL_SURFACE | 0.0593 | 0.123541667 |
| HALLMARK_UV_RESPONSE_UP | 0.0726 | 0.1452 |
| HALLMARK_MTORC1_SIGNALING | 0.0827 | 0.159038462 |
| HALLMARK_ALLOGRAFT_REJECTION | 0.2044 | 0.378518519 |
| HALLMARK_DNA_REPAIR | 0.225 | 0.401785714 |
| HALLMARK_HEME_METABOLISM | 0.2687 | 0.449193548 |
| HALLMARK_PEROXISOME | 0.2711 | 0.449193548 |
| HALLMARK_TGF_BETA_SIGNALING | 0.2785 | 0.449193548 |
| HALLMARK_BILE_ACID_METABOLISM | 0.3176 | 0.49625 |
| HALLMARK_MYC_TARGETS_V2 | 0.376 | 0.56969697 |
| HALLMARK_CHOLESTEROL_HOMEOSTASIS | 0.4723 | 0.694558824 |
| HALLMARK_PROTEIN_SECRETION | 0.5884 | 0.840571429 |
| HALLMARK_ANDROGEN_RESPONSE | 0.6312 | 0.857837838 |
| HALLMARK_WNT_BETA_CATENIN_SIGNALING | 0.6348 | 0.857837838 |
| HALLMARK_SPERMATOGENESIS | 0.6707 | 0.8825 |
| HALLMARK_REACTIVE_OXIGEN_SPECIES_PATHWAY | 0.7166 | 0.896875 |
| HALLMARK_G2M_CHECKPOINT | 0.7175 | 0.896875 |
| HALLMARK_ADIPOGENESIS | 0.7542 | 0.919756098 |
| HALLMARK_XENOBIOTIC_METABOLISM | 0.8072 | 0.952790698 |
| HALLMARK_KRAS_SIGNALING_UP | 0.8194 | 0.952790698 |
| HALLMARK_COAGULATION | 0.9334 | 1 |
| HALLMARK_ANGIOGENESIS | 0.9352 | 1 |
| HALLMARK_PANCREAS_BETA_CELLS | 0.9707 | 1 |
| HALLMARK_FATTY_ACID_METABOLISM | 0.9777 | 1 |
| HALLMARK_E2F_TARGETS | 0.9846 | 1 |
| HALLMARK_OXIDATIVE_PHOSPHORYLATION | 0.9996 | 1 |
| HALLMARK_MYC_TARGETS_V1 | 1 | 1 |

**Supplementary file 1g |** Gene sets decreased with age in encapsulated hskMPs compared to the young control after ten days *in vivo*. Data are derived from capsules of n≥5 mice in each age group. GST Pval = Wilcoxon gene set test p-value. GST FDR = Adjusted p-value using the Benjamini-Hochberg procedure.

| **Gene Set Name - Mouse** | **GST Pval** | **GST FDR** |
| --- | --- | --- |
| HALLMARK_XENOBIOTIC_METABOLISM | 1.00E-04 | 0.001 |
| HALLMARK_P53_PATHWAY | 1.00E-04 | 0.001 |
| HALLMARK_COAGULATION | 1.00E-04 | 0.001 |
| HALLMARK_IL2_STAT5_SIGNALING | 1.00E-04 | 0.001 |
| HALLMARK_KRAS_SIGNALING_UP | 1.00E-04 | 0.001 |
| HALLMARK_COMPLEMENT | 8.00E-04 | 0.006666667 |
| HALLMARK_ADIPOGENESIS | 0.0018 | 0.012857143 |
| HALLMARK_IL6_JAK_STAT3_SIGNALING | 0.0036 | 0.0225 |
| HALLMARK_REACTIVE_OXIGEN_SPECIES_PATHWAY | 0.0043 | 0.023888889 |
| HALLMARK_ANGIOGENESIS | 0.0089 | 0.0445 |
| HALLMARK_INFLAMMATORY_RESPONSE | 0.0296 | 0.134545455 |
| HALLMARK_INTERFERON_GAMMA_RESPONSE | 0.0348 | 0.145 |
| HALLMARK_HEME_METABOLISM | 0.0391 | 0.150384615 |
| HALLMARK_ALLOGRAFT_REJECTION | 0.0561 | 0.198823529 |
| HALLMARK_PANCREAS_BETA_CELLS | 0.0606 | 0.198823529 |
| HALLMARK_BILE_ACID_METABOLISM | 0.0648 | 0.198823529 |
| HALLMARK_MYOGENESIS | 0.0676 | 0.198823529 |
| HALLMARK_PROTEIN_SECRETION | 0.0918 | 0.255 |
| HALLMARK_EPITHELIAL_MESENCHYMAL_TRANSITION | 0.1071 | 0.268809524 |
| HALLMARK_ESTROGEN_RESPONSE_EARLY | 0.1125 | 0.268809524 |
| HALLMARK_GLYCOLYSIS | 0.1129 | 0.268809524 |
| HALLMARK_UV_RESPONSE_UP | 0.1237 | 0.281136364 |
| HALLMARK_DNA_REPAIR | 0.1388 | 0.30173913 |
| HALLMARK_WNT_BETA_CATENIN_SIGNALING | 0.1468 | 0.305833333 |
| HALLMARK_HYPOXIA | 0.1736 | 0.334038462 |
| HALLMARK_PEROXISOME | 0.1737 | 0.334038462 |
| HALLMARK_APOPTOSIS | 0.183 | 0.33625 |
| HALLMARK_UNFOLDED_PROTEIN_RESPONSE | 0.1883 | 0.33625 |
| HALLMARK_INTERFERON_ALPHA_RESPONSE | 0.2077 | 0.3485 |
| HALLMARK_APICAL_SURFACE | 0.2091 | 0.3485 |
| HALLMARK_TNFA_SIGNALING_VIA_NFKB | 0.2938 | 0.473870968 |
| HALLMARK_SPERMATOGENESIS | 0.3287 | 0.51359375 |
| HALLMARK_ESTROGEN_RESPONSE_LATE | 0.3518 | 0.533030303 |
| HALLMARK_MTORC1_SIGNALING | 0.3919 | 0.576323529 |
| HALLMARK_FATTY_ACID_METABOLISM | 0.4534 | 0.640555556 |
| HALLMARK_OXIDATIVE_PHOSPHORYLATION | 0.4612 | 0.640555556 |
| HALLMARK_ANDROGEN_RESPONSE | 0.5669 | 0.766081081 |
| HALLMARK_PI3K_AKT_MTOR_SIGNALING | 0.5979 | 0.768333333 |
| HALLMARK_APICAL_JUNCTION | 0.5993 | 0.768333333 |
| HALLMARK_TGF_BETA_SIGNALING | 0.7692 | 0.9615 |
| HALLMARK_NOTCH_SIGNALING | 0.8402 | 1 |
| HALLMARK_CHOLESTEROL_HOMEOSTASIS | 0.873 | 1 |
| HALLMARK_KRAS_SIGNALING_DN | 0.8786 | 1 |
| HALLMARK_HEDGEHOG_SIGNALING | 0.9466 | 1 |
| HALLMARK_UV_RESPONSE_DN | 0.9712 | 1 |
| HALLMARK_MYC_TARGETS_V2 | 0.9741 | 1 |
| HALLMARK_MITOTIC_SPINDLE | 1 | 1 |
| HALLMARK_G2M_CHECKPOINT | 1 | 1 |
| HALLMARK_E2F_TARGETS | 1 | 1 |
| HALLMARK_MYC_TARGETS_V1 | 1 | 1 |

**Supplementary file 1h** | Gene sets decreased with age in encapsulated mskMPs compared to the young control after ten days *in vivo*. Data are derived from capsules of n≥5 mice in each age group. GST Pval = Wilcoxon gene set test p-value. GST FDR = Adjusted p-value using the Benjamini-Hochberg procedure.

| **Gene Set Name - Freshly Isolated mouse muscle stem cells** | **GST Pval** | **GST FDR** |
| --- | --- | --- |
| HALLMARK_TNFA_SIGNALING_VIA_NFKB | 1.00E-04 | 0.000714286 |
| HALLMARK_INTERFERON_ALPHA_RESPONSE | 1.00E-04 | 0.000714286 |
| HALLMARK_INTERFERON_GAMMA_RESPONSE | 1.00E-04 | 0.000714286 |
| HALLMARK_E2F_TARGETS | 1.00E-04 | 0.000714286 |
| HALLMARK_XENOBIOTIC_METABOLISM | 1.00E-04 | 0.000714286 |
| HALLMARK_FATTY_ACID_METABOLISM | 1.00E-04 | 0.000714286 |
| HALLMARK_ALLOGRAFT_REJECTION | 1.00E-04 | 0.000714286 |
| HALLMARK_MTORC1_SIGNALING | 2.00E-04 | 0.001111111 |
| HALLMARK_MYC_TARGETS_V2 | 2.00E-04 | 0.001111111 |
| HALLMARK_OXIDATIVE_PHOSPHORYLATION | 4.00E-04 | 0.002 |
| HALLMARK_G2M_CHECKPOINT | 8.00E-04 | 0.003636364 |
| HALLMARK_IL6_JAK_STAT3_SIGNALING | 0.0028 | 0.011153846 |
| HALLMARK_INFLAMMATORY_RESPONSE | 0.0029 | 0.011153846 |
| HALLMARK_REACTIVE_OXIGEN_SPECIES_PATHWAY | 0.0039 | 0.013928571 |
| HALLMARK_MYC_TARGETS_V1 | 0.0047 | 0.015666667 |
| HALLMARK_APOPTOSIS | 0.0074 | 0.023125 |
| HALLMARK_BILE_ACID_METABOLISM | 0.0194 | 0.057058824 |
| HALLMARK_KRAS_SIGNALING_UP | 0.0265 | 0.073611111 |
| HALLMARK_COMPLEMENT | 0.0371 | 0.097631579 |
| HALLMARK_DNA_REPAIR | 0.0452 | 0.113 |
| HALLMARK_PEROXISOME | 0.0858 | 0.204285714 |
| HALLMARK_SPERMATOGENESIS | 0.1062 | 0.241363636 |
| HALLMARK_GLYCOLYSIS | 0.1574 | 0.342173913 |
| HALLMARK_APICAL_SURFACE | 0.2034 | 0.42375 |
| HALLMARK_ESTROGEN_RESPONSE_LATE | 0.2152 | 0.4304 |
| HALLMARK_IL2_STAT5_SIGNALING | 0.2275 | 0.4375 |
| HALLMARK_ADIPOGENESIS | 0.2721 | 0.503888889 |
| HALLMARK_CHOLESTEROL_HOMEOSTASIS | 0.2845 | 0.508035714 |
| HALLMARK_UNFOLDED_PROTEIN_RESPONSE | 0.326 | 0.562068966 |
| HALLMARK_PANCREAS_BETA_CELLS | 0.39 | 0.65 |
| HALLMARK_HEME_METABOLISM | 0.4151 | 0.65890625 |
| HALLMARK_COAGULATION | 0.4217 | 0.65890625 |
| HALLMARK_P53_PATHWAY | 0.4672 | 0.707878788 |
| HALLMARK_HYPOXIA | 0.4937 | 0.726029412 |
| HALLMARK_ANDROGEN_RESPONSE | 0.556 | 0.794285714 |
| HALLMARK_PI3K_AKT_MTOR_SIGNALING | 0.5926 | 0.823055556 |
| HALLMARK_UV_RESPONSE_UP | 0.7824 | 1 |
| HALLMARK_ESTROGEN_RESPONSE_EARLY | 0.8029 | 1 |
| HALLMARK_WNT_BETA_CATENIN_SIGNALING | 0.8277 | 1 |
| HALLMARK_PROTEIN_SECRETION | 0.841 | 1 |
| HALLMARK_TGF_BETA_SIGNALING | 0.9167 | 1 |
| HALLMARK_NOTCH_SIGNALING | 0.9188 | 1 |
| HALLMARK_HEDGEHOG_SIGNALING | 0.9471 | 1 |
| HALLMARK_MITOTIC_SPINDLE | 0.9955 | 1 |
| HALLMARK_KRAS_SIGNALING_DN | 0.9984 | 1 |
| HALLMARK_MYOGENESIS | 0.9994 | 1 |
| HALLMARK_APICAL_JUNCTION | 0.9996 | 1 |
| HALLMARK_UV_RESPONSE_DN | 0.9999 | 1 |
| HALLMARK_EPITHELIAL_MESENCHYMAL_TRANSITION | 1 | 1 |
| HALLMARK_ANGIOGENESIS | 1 | 1 |

**Supplementary file 1i |** Gene sets increased with age in niche resident freshly isolated mouse muscle stem cells compared to the young control. Data from n=8 young or aged mice. GST Pval = Wilcoxon gene set test p-value. GST FDR = Adjusted p-value using the Benjamini-Hochberg procedure.

| **Gene Set Name - Freshly Isolated mouse muscle stem cells** | **GST Pval** | **GST FDR** |
| --- | --- | --- |
| HALLMARK_EPITHELIAL_MESENCHYMAL_TRANSITION | 1.00E-04 | 0.001666667 |
| HALLMARK_UV_RESPONSE_DN | 1.00E-04 | 0.001666667 |
| HALLMARK_ANGIOGENESIS | 1.00E-04 | 0.001666667 |
| HALLMARK_MYOGENESIS | 2.00E-04 | 0.0025 |
| HALLMARK_APICAL_JUNCTION | 4.00E-04 | 0.004 |
| HALLMARK_KRAS_SIGNALING_DN | 0.0019 | 0.015833333 |
| HALLMARK_MITOTIC_SPINDLE | 0.0055 | 0.039285714 |
| HALLMARK_HEDGEHOG_SIGNALING | 0.0509 | 0.318125 |
| HALLMARK_NOTCH_SIGNALING | 0.0785 | 0.435 |
| HALLMARK_TGF_BETA_SIGNALING | 0.087 | 0.435 |
| HALLMARK_PROTEIN_SECRETION | 0.1603 | 0.728636364 |
| HALLMARK_WNT_BETA_CATENIN_SIGNALING | 0.1808 | 0.739615385 |
| HALLMARK_ESTROGEN_RESPONSE_EARLY | 0.1923 | 0.739615385 |
| HALLMARK_UV_RESPONSE_UP | 0.2166 | 0.773571429 |
| HALLMARK_PI3K_AKT_MTOR_SIGNALING | 0.3975 | 1 |
| HALLMARK_ANDROGEN_RESPONSE | 0.4511 | 1 |
| HALLMARK_HYPOXIA | 0.5136 | 1 |
| HALLMARK_P53_PATHWAY | 0.5311 | 1 |
| HALLMARK_COAGULATION | 0.585 | 1 |
| HALLMARK_HEME_METABOLISM | 0.5856 | 1 |
| HALLMARK_PANCREAS_BETA_CELLS | 0.6043 | 1 |
| HALLMARK_UNFOLDED_PROTEIN_RESPONSE | 0.667 | 1 |
| HALLMARK_CHOLESTEROL_HOMEOSTASIS | 0.7074 | 1 |
| HALLMARK_ADIPOGENESIS | 0.7445 | 1 |
| HALLMARK_IL2_STAT5_SIGNALING | 0.768 | 1 |
| HALLMARK_ESTROGEN_RESPONSE_LATE | 0.7814 | 1 |
| HALLMARK_APICAL_SURFACE | 0.8004 | 1 |
| HALLMARK_GLYCOLYSIS | 0.8457 | 1 |
| HALLMARK_SPERMATOGENESIS | 0.8918 | 1 |
| HALLMARK_PEROXISOME | 0.9184 | 1 |
| HALLMARK_DNA_REPAIR | 0.9581 | 1 |
| HALLMARK_COMPLEMENT | 0.9625 | 1 |
| HALLMARK_KRAS_SIGNALING_UP | 0.9756 | 1 |
| HALLMARK_BILE_ACID_METABOLISM | 0.9823 | 1 |
| HALLMARK_APOPTOSIS | 0.9911 | 1 |
| HALLMARK_REACTIVE_OXIGEN_SPECIES_PATHWAY | 0.9948 | 1 |
| HALLMARK_MYC_TARGETS_V1 | 0.9957 | 1 |
| HALLMARK_IL6_JAK_STAT3_SIGNALING | 0.9977 | 1 |
| HALLMARK_INFLAMMATORY_RESPONSE | 0.9982 | 1 |
| HALLMARK_MTORC1_SIGNALING | 0.9998 | 1 |
| HALLMARK_G2M_CHECKPOINT | 0.9999 | 1 |
| HALLMARK_FATTY_ACID_METABOLISM | 0.9999 | 1 |
| HALLMARK_TNFA_SIGNALING_VIA_NFKB | 1 | 1 |
| HALLMARK_INTERFERON_ALPHA_RESPONSE | 1 | 1 |
| HALLMARK_INTERFERON_GAMMA_RESPONSE | 1 | 1 |
| HALLMARK_E2F_TARGETS | 1 | 1 |
| HALLMARK_MYC_TARGETS_V2 | 1 | 1 |
| HALLMARK_XENOBIOTIC_METABOLISM | 1 | 1 |
| HALLMARK_OXIDATIVE_PHOSPHORYLATION | 1 | 1 |
| HALLMARK_ALLOGRAFT_REJECTION | 1 | 1 |

**Supplementary file 1j |** Gene sets decreased with age in niche resident freshly isolated mouse muscle stem cells compared to the young control. Data from n=3 young or aged mice. GST Pval = Wilcoxon gene set test p-value. GST FDR = Adjusted p-value using the Benjamini-Hochberg procedure.

| **Gene Set Name: Human - D4 - *in-vitro*** | **GST_Pval** | **GST_FDR** |
| --- | --- | --- |
| HALLMARK_KRAS_SIGNALING_DN | 7.38184E-05 | 0.003690921 |
| HALLMARK_MYOGENESIS | 0.016547233 | 0.413680814 |
| HALLMARK_MITOTIC_SPINDLE | 0.092710962 | 1 |
| HALLMARK_WNT_BETA_CATENIN_SIGNALING | 0.127484809 | 1 |
| HALLMARK_SPERMATOGENESIS | 0.155043207 | 1 |
| HALLMARK_HEDGEHOG_SIGNALING | 0.308988817 | 1 |
| HALLMARK_KRAS_SIGNALING_UP | 0.489306906 | 1 |
| HALLMARK_UV_RESPONSE_DN | 0.502201637 | 1 |
| HALLMARK_NOTCH_SIGNALING | 0.523833904 | 1 |
| HALLMARK_G2M_CHECKPOINT | 0.629324403 | 1 |
| HALLMARK_APICAL_SURFACE | 0.754826941 | 1 |
| HALLMARK_E2F_TARGETS | 0.780390542 | 1 |
| HALLMARK_ANGIOGENESIS | 0.866321537 | 1 |
| HALLMARK_INTERFERON_ALPHA_RESPONSE | 0.909711872 | 1 |
| HALLMARK_INFLAMMATORY_RESPONSE | 0.918050427 | 1 |
| HALLMARK_APICAL_JUNCTION | 0.919415481 | 1 |
| HALLMARK_PANCREAS_BETA_CELLS | 0.940161163 | 1 |
| HALLMARK_IL2_STAT5_SIGNALING | 0.960055152 | 1 |
| HALLMARK_TNFA_SIGNALING_VIA_NFKB | 0.966177046 | 1 |
| HALLMARK_INTERFERON_GAMMA_RESPONSE | 0.967155442 | 1 |
| HALLMARK_TGF_BETA_SIGNALING | 0.972137076 | 1 |
| HALLMARK_IL6_JAK_STAT3_SIGNALING | 0.983257911 | 1 |
| HALLMARK_MYC_TARGETS_V2 | 0.988243344 | 1 |
| HALLMARK_BILE_ACID_METABOLISM | 0.993171096 | 1 |
| HALLMARK_COMPLEMENT | 0.995493526 | 1 |
| HALLMARK_DNA_REPAIR | 0.996835177 | 1 |
| HALLMARK_UNFOLDED_PROTEIN_RESPONSE | 0.996925414 | 1 |
| HALLMARK_UV_RESPONSE_UP | 0.998857485 | 1 |
| HALLMARK_ANDROGEN_RESPONSE | 0.999000297 | 1 |
| HALLMARK_COAGULATION | 0.999724498 | 1 |
| HALLMARK_PI3K_AKT_MTOR_SIGNALING | 0.999749468 | 1 |
| HALLMARK_P53_PATHWAY | 0.99977587 | 1 |
| HALLMARK_ESTROGEN_RESPONSE_EARLY | 0.999855771 | 1 |
| HALLMARK_ESTROGEN_RESPONSE_LATE | 0.999880179 | 1 |
| HALLMARK_ALLOGRAFT_REJECTION | 0.999921122 | 1 |
| HALLMARK_PEROXISOME | 0.99993053 | 1 |
| HALLMARK_HEME_METABOLISM | 0.999989611 | 1 |
| HALLMARK_PROTEIN_SECRETION | 0.999993718 | 1 |
| HALLMARK_APOPTOSIS | 0.999996336 | 1 |
| HALLMARK_CHOLESTEROL_HOMEOSTASIS | 0.999998683 | 1 |
| HALLMARK_XENOBIOTIC_METABOLISM | 0.999999212 | 1 |
| HALLMARK_HYPOXIA | 0.999999372 | 1 |
| HALLMARK_GLYCOLYSIS | 0.999999863 | 1 |
| HALLMARK_REACTIVE_OXIGEN_SPECIES_PATHWAY | 0.999999898 | 1 |
| HALLMARK_EPITHELIAL_MESENCHYMAL_TRANSITION | 1 | 1 |
| HALLMARK_ADIPOGENESIS | 1 | 1 |
| HALLMARK_FATTY_ACID_METABOLISM | 1 | 1 |
| HALLMARK_MTORC1_SIGNALING | 1 | 1 |
| HALLMARK_MYC_TARGETS_V1 | 1 | 1 |
| HALLMARK_OXIDATIVE_PHOSPHORYLATION | 1 | 1 |

**Supplementary file 1k |** Gene sets increased after four days in 2D hskMP culture exposed to aged human serum compared to the young human serum. GST Pval = Wilcoxon gene set test p-value. Data are derived from hskMPs exposed to human serum from n=3 different young or aged donors. GST FDR = Adjusted p-value using the Benjamini-Hochberg procedure.

| **Gene Set Name: Human - D10 - *in-vitro*** | **GST_Pval** | **GST_FDR** |
| --- | --- | --- |
| HALLMARK_OXIDATIVE_PHOSPHORYLATION | 2.36115E-07 | 1.18058E-05 |
| HALLMARK_MYC_TARGETS_V1 | 0.000955568 | 0.0238892 |
| HALLMARK_E2F_TARGETS | 0.003459696 | 0.057661598 |
| HALLMARK_G2M_CHECKPOINT | 0.00849323 | 0.106165371 |
| HALLMARK_MYC_TARGETS_V2 | 0.029563729 | 0.295637295 |
| HALLMARK_MYOGENESIS | 0.074208319 | 0.618402659 |
| HALLMARK_MITOTIC_SPINDLE | 0.193517125 | 0.999999997 |
| HALLMARK_SPERMATOGENESIS | 0.237153025 | 0.999999997 |
| HALLMARK_ADIPOGENESIS | 0.246304701 | 0.999999997 |
| HALLMARK_DNA_REPAIR | 0.307501786 | 0.999999997 |
| HALLMARK_WNT_BETA_CATENIN_SIGNALING | 0.318443285 | 0.999999997 |
| HALLMARK_NOTCH_SIGNALING | 0.377910729 | 0.999999997 |
| HALLMARK_BILE_ACID_METABOLISM | 0.413454301 | 0.999999997 |
| HALLMARK_HEME_METABOLISM | 0.481253969 | 0.999999997 |
| HALLMARK_UV_RESPONSE_DN | 0.52024193 | 0.999999997 |
| HALLMARK_PI3K_AKT_MTOR_SIGNALING | 0.58492227 | 0.999999997 |
| HALLMARK_HEDGEHOG_SIGNALING | 0.58804766 | 0.999999997 |
| HALLMARK_KRAS_SIGNALING_DN | 0.605869514 | 0.999999997 |
| HALLMARK_ANDROGEN_RESPONSE | 0.748988541 | 0.999999997 |
| HALLMARK_PEROXISOME | 0.803261712 | 0.999999997 |
| HALLMARK_GLYCOLYSIS | 0.821549992 | 0.999999997 |
| HALLMARK_UNFOLDED_PROTEIN_RESPONSE | 0.859727268 | 0.999999997 |
| HALLMARK_FATTY_ACID_METABOLISM | 0.862474641 | 0.999999997 |
| HALLMARK_PROTEIN_SECRETION | 0.869307435 | 0.999999997 |
| HALLMARK_ALLOGRAFT_REJECTION | 0.886954491 | 0.999999997 |
| HALLMARK_P53_PATHWAY | 0.904334185 | 0.999999997 |
| HALLMARK_ANGIOGENESIS | 0.909348451 | 0.999999997 |
| HALLMARK_IL6_JAK_STAT3_SIGNALING | 0.914068939 | 0.999999997 |
| HALLMARK_REACTIVE_OXIGEN_SPECIES_PATHWAY | 0.922475651 | 0.999999997 |
| HALLMARK_APICAL_SURFACE | 0.928490118 | 0.999999997 |
| HALLMARK_IL2_STAT5_SIGNALING | 0.959439771 | 0.999999997 |
| HALLMARK_UV_RESPONSE_UP | 0.967586043 | 0.999999997 |
| HALLMARK_ESTROGEN_RESPONSE_LATE | 0.972020235 | 0.999999997 |
| HALLMARK_XENOBIOTIC_METABOLISM | 0.979705067 | 0.999999997 |
| HALLMARK_COMPLEMENT | 0.99032617 | 0.999999997 |
| HALLMARK_ESTROGEN_RESPONSE_EARLY | 0.990701043 | 0.999999997 |
| HALLMARK_PANCREAS_BETA_CELLS | 0.991640362 | 0.999999997 |
| HALLMARK_KRAS_SIGNALING_UP | 0.992024922 | 0.999999997 |
| HALLMARK_TGF_BETA_SIGNALING | 0.99293276 | 0.999999997 |
| HALLMARK_INFLAMMATORY_RESPONSE | 0.995120988 | 0.999999997 |
| HALLMARK_HYPOXIA | 0.998285232 | 0.999999997 |
| HALLMARK_APICAL_JUNCTION | 0.998711642 | 0.999999997 |
| HALLMARK_TNFA_SIGNALING_VIA_NFKB | 0.998906247 | 0.999999997 |
| HALLMARK_INTERFERON_ALPHA_RESPONSE | 0.99980783 | 0.999999997 |
| HALLMARK_INTERFERON_GAMMA_RESPONSE | 0.999847467 | 0.999999997 |
| HALLMARK_APOPTOSIS | 0.999915392 | 0.999999997 |
| HALLMARK_MTORC1_SIGNALING | 0.999986277 | 0.999999997 |
| HALLMARK_COAGULATION | 0.999991192 | 0.999999997 |
| HALLMARK_EPITHELIAL_MESENCHYMAL_TRANSITION | 0.999999972 | 0.999999997 |
| HALLMARK_CHOLESTEROL_HOMEOSTASIS | 0.999999997 | 0.999999997 |

**Supplementary file 1l |** Gene sets increased after ten days in 2D hskMP culture exposed to aged human serum compared to the young human serum. Data are derived from hskMPs exposed to human serum from n=3 different young or aged donors. GST Pval = Wilcoxon gene set test p-value. GST FDR = Adjusted p-value using the Benjamini-Hochberg procedure.

| **Gene Set Name: Human - D4 - *in-vitro*** | **GST_Pval** | **GST_FDR** |
| --- | --- | --- |
| HALLMARK_OXIDATIVE_PHOSPHORYLATION | 2.37546E-27 | 1.18773E-25 |
| HALLMARK_MYC_TARGETS_V1 | 9.99263E-21 | 2.49816E-19 |
| HALLMARK_MTORC1_SIGNALING | 1.85244E-17 | 3.0874E-16 |
| HALLMARK_FATTY_ACID_METABOLISM | 4.28616E-13 | 5.3577E-12 |
| HALLMARK_ADIPOGENESIS | 7.65179E-12 | 7.65179E-11 |
| HALLMARK_EPITHELIAL_MESENCHYMAL_TRANSITION | 1.62655E-10 | 1.35546E-09 |
| HALLMARK_REACTIVE_OXIGEN_SPECIES_PATHWAY | 1.02231E-07 | 7.30222E-07 |
| HALLMARK_GLYCOLYSIS | 1.36731E-07 | 8.54569E-07 |
| HALLMARK_HYPOXIA | 6.2757E-07 | 3.4865E-06 |
| HALLMARK_XENOBIOTIC_METABOLISM | 7.87998E-07 | 3.93999E-06 |
| HALLMARK_CHOLESTEROL_HOMEOSTASIS | 1.31717E-06 | 5.98715E-06 |
| HALLMARK_APOPTOSIS | 3.66435E-06 | 1.52681E-05 |
| HALLMARK_PROTEIN_SECRETION | 6.28291E-06 | 2.4165E-05 |
| HALLMARK_HEME_METABOLISM | 1.03897E-05 | 3.71061E-05 |
| HALLMARK_PEROXISOME | 6.94786E-05 | 0.000231595 |
| HALLMARK_ALLOGRAFT_REJECTION | 7.88859E-05 | 0.000246518 |
| HALLMARK_ESTROGEN_RESPONSE_LATE | 0.000119831 | 0.000352445 |
| HALLMARK_ESTROGEN_RESPONSE_EARLY | 0.000144241 | 0.000400668 |
| HALLMARK_P53_PATHWAY | 0.000224146 | 0.000589858 |
| HALLMARK_PI3K_AKT_MTOR_SIGNALING | 0.000250558 | 0.000626395 |
| HALLMARK_COAGULATION | 0.000275532 | 0.000656028 |
| HALLMARK_ANDROGEN_RESPONSE | 0.0009998 | 0.002272272 |
| HALLMARK_UV_RESPONSE_UP | 0.0011426 | 0.002483913 |
| HALLMARK_UNFOLDED_PROTEIN_RESPONSE | 0.003074825 | 0.00633007 |
| HALLMARK_DNA_REPAIR | 0.003165035 | 0.00633007 |
| HALLMARK_COMPLEMENT | 0.004506768 | 0.008666861 |
| HALLMARK_BILE_ACID_METABOLISM | 0.00682946 | 0.012647149 |
| HALLMARK_MYC_TARGETS_V2 | 0.011757726 | 0.020995939 |
| HALLMARK_IL6_JAK_STAT3_SIGNALING | 0.016743553 | 0.028868194 |
| HALLMARK_TGF_BETA_SIGNALING | 0.027865236 | 0.04644206 |
| HALLMARK_INTERFERON_GAMMA_RESPONSE | 0.032846143 | 0.052850754 |
| HALLMARK_TNFA_SIGNALING_VIA_NFKB | 0.033824482 | 0.052850754 |
| HALLMARK_IL2_STAT5_SIGNALING | 0.039946673 | 0.060525262 |
| HALLMARK_PANCREAS_BETA_CELLS | 0.059846482 | 0.088009533 |
| HALLMARK_APICAL_JUNCTION | 0.080587632 | 0.113823879 |
| HALLMARK_INFLAMMATORY_RESPONSE | 0.081953193 | 0.113823879 |
| HALLMARK_INTERFERON_ALPHA_RESPONSE | 0.090292878 | 0.122017402 |
| HALLMARK_ANGIOGENESIS | 0.133689274 | 0.175906939 |
| HALLMARK_E2F_TARGETS | 0.219615085 | 0.281557801 |
| HALLMARK_APICAL_SURFACE | 0.245188044 | 0.306485055 |
| HALLMARK_G2M_CHECKPOINT | 0.37068282 | 0.452052219 |
| HALLMARK_NOTCH_SIGNALING | 0.476186059 | 0.566888165 |
| HALLMARK_UV_RESPONSE_DN | 0.497807442 | 0.578845863 |
| HALLMARK_KRAS_SIGNALING_UP | 0.51070201 | 0.580343194 |
| HALLMARK_HEDGEHOG_SIGNALING | 0.691028537 | 0.767809485 |
| HALLMARK_SPERMATOGENESIS | 0.844964205 | 0.918439353 |
| HALLMARK_WNT_BETA_CATENIN_SIGNALING | 0.87252442 | 0.928217468 |
| HALLMARK_MITOTIC_SPINDLE | 0.907292211 | 0.945096053 |
| HALLMARK_MYOGENESIS | 0.983453569 | 0.99992619 |
| HALLMARK_KRAS_SIGNALING_DN | 0.99992619 | 0.99992619 |

**Supplementary file 1m |** Gene sets decreased after four days in 2D hskMP culture exposed to aged human serum compared to the young human serum. Data are derived from hskMPs exposed to human serum from n=3 different young or aged donors. GST Pval = Wilcoxon gene set test p-value. GST FDR = Adjusted p-value using the Benjamini-Hochberg procedure.

| **Gene Set Name: Human - D10 - *in-vitro*** | **GST_Pval** | **GST_FDR** |
| --- | --- | --- |
| HALLMARK_CHOLESTEROL_HOMEOSTASIS | 3.38115E-09 | 1.69057E-07 |
| HALLMARK_EPITHELIAL_MESENCHYMAL_TRANSITION | 2.8198E-08 | 7.0495E-07 |
| HALLMARK_COAGULATION | 8.80894E-06 | 0.000146816 |
| HALLMARK_MTORC1_SIGNALING | 1.37243E-05 | 0.000171553 |
| HALLMARK_APOPTOSIS | 8.46158E-05 | 0.000846158 |
| HALLMARK_INTERFERON_GAMMA_RESPONSE | 0.000152545 | 0.001271212 |
| HALLMARK_INTERFERON_ALPHA_RESPONSE | 0.000192191 | 0.001372792 |
| HALLMARK_TNFA_SIGNALING_VIA_NFKB | 0.001093828 | 0.006836424 |
| HALLMARK_APICAL_JUNCTION | 0.001288447 | 0.007158037 |
| HALLMARK_HYPOXIA | 0.001714879 | 0.008574394 |
| HALLMARK_INFLAMMATORY_RESPONSE | 0.00487935 | 0.022178864 |
| HALLMARK_TGF_BETA_SIGNALING | 0.00706795 | 0.029449793 |
| HALLMARK_KRAS_SIGNALING_UP | 0.007975567 | 0.029861084 |
| HALLMARK_PANCREAS_BETA_CELLS | 0.008361104 | 0.029861084 |
| HALLMARK_ESTROGEN_RESPONSE_EARLY | 0.009299486 | 0.030232521 |
| HALLMARK_COMPLEMENT | 0.009674407 | 0.030232521 |
| HALLMARK_XENOBIOTIC_METABOLISM | 0.020296026 | 0.059694193 |
| HALLMARK_ESTROGEN_RESPONSE_LATE | 0.027981149 | 0.077725414 |
| HALLMARK_UV_RESPONSE_UP | 0.032415584 | 0.085304169 |
| HALLMARK_IL2_STAT5_SIGNALING | 0.040562078 | 0.101405195 |
| HALLMARK_APICAL_SURFACE | 0.071516386 | 0.170277109 |
| HALLMARK_REACTIVE_OXIGEN_SPECIES_PATHWAY | 0.07752997 | 0.176204476 |
| HALLMARK_IL6_JAK_STAT3_SIGNALING | 0.085936579 | 0.186818651 |
| HALLMARK_ANGIOGENESIS | 0.090659734 | 0.188874445 |
| HALLMARK_P53_PATHWAY | 0.095669122 | 0.191338244 |
| HALLMARK_ALLOGRAFT_REJECTION | 0.113050418 | 0.217404651 |
| HALLMARK_PROTEIN_SECRETION | 0.130698452 | 0.241859331 |
| HALLMARK_FATTY_ACID_METABOLISM | 0.137530456 | 0.241859331 |
| HALLMARK_UNFOLDED_PROTEIN_RESPONSE | 0.140278412 | 0.241859331 |
| HALLMARK_GLYCOLYSIS | 0.178455331 | 0.297425551 |
| HALLMARK_PEROXISOME | 0.196746281 | 0.317332711 |
| HALLMARK_ANDROGEN_RESPONSE | 0.251020589 | 0.39221967 |
| HALLMARK_KRAS_SIGNALING_DN | 0.394140878 | 0.592983711 |
| HALLMARK_HEDGEHOG_SIGNALING | 0.411971511 | 0.592983711 |
| HALLMARK_PI3K_AKT_MTOR_SIGNALING | 0.415088598 | 0.592983711 |
| HALLMARK_UV_RESPONSE_DN | 0.479767137 | 0.666343246 |
| HALLMARK_HEME_METABOLISM | 0.518754536 | 0.701019643 |
| HALLMARK_BILE_ACID_METABOLISM | 0.586557064 | 0.77178561 |
| HALLMARK_NOTCH_SIGNALING | 0.622108325 | 0.797574776 |
| HALLMARK_WNT_BETA_CATENIN_SIGNALING | 0.681572497 | 0.844519472 |
| HALLMARK_DNA_REPAIR | 0.692505967 | 0.844519472 |
| HALLMARK_ADIPOGENESIS | 0.753701485 | 0.887042534 |
| HALLMARK_SPERMATOGENESIS | 0.76285658 | 0.887042534 |
| HALLMARK_MITOTIC_SPINDLE | 0.806488121 | 0.916463774 |
| HALLMARK_MYOGENESIS | 0.925794414 | 0.999999764 |
| HALLMARK_MYC_TARGETS_V2 | 0.970438614 | 0.999999764 |
| HALLMARK_G2M_CHECKPOINT | 0.991507212 | 0.999999764 |
| HALLMARK_E2F_TARGETS | 0.996540502 | 0.999999764 |
| HALLMARK_MYC_TARGETS_V1 | 0.999044493 | 0.999999764 |
| HALLMARK_OXIDATIVE_PHOSPHORYLATION | 0.999999764 | 0.999999764 |

**Supplementary file 1n |** Gene sets decreased after ten days in 2D hskMP culture exposed to aged human serum compared to the young human serum. Data are derived from hskMPs exposed to human serum from n=3 different young or aged donors. GST Pval = Wilcoxon gene set test p-value. GST FDR = Adjusted p-value using the Benjamini-Hochberg procedure.
